# Patent ductus arterious and increased conjugated bilirubin in the second week after birth are independent risk factors for necrotizing enterocolitis in preterm infants: an observational study

**DOI:** 10.1186/s12887-023-04173-0

**Published:** 2023-07-13

**Authors:** Xiaoya Han, Shudong Cui

**Affiliations:** 1 Department of Pediatrics, The Fourth Hospital of Hebei Medical University, Shijiazhuang, China; 2 Department of Pediatrics, The First Affiliated Hospital of Nanjing Medical University, Nanjing, China

**Keywords:** Neonatal necrotizing enterocolitis, Conjugated bilirubin, Preterm infants

## Abstract

**Background:**

Neonatal necrotizing enterocolitis (NEC) is a common critical illness of the gastrointestinal system in neonatal intensive care units with complex causes. We want to explore effects of serum-conjugated bilirubin on the occurrence of NEC in preterm infants.

**Methods:**

A retrospective study of clinical case data of premature infants from 2017 to 2020 in the Department of pediatrics of the First Affiliated Hospital of Nanjing Medical University was conducted. Among these, 41 were diagnosed with NEC. After screening, 2 cases were excluded because of incomplete data. Propensity-matching score (PSM) was performed according to the ratio of 1:2(2 preterm infants in the NEC group were not matched), and finally, 37 cases were in the NEC group (average time to diagnosis was 18.9 days), and 74 cases in the non-NEC group. We compared the difference between the NEC and non-NEC groups in early serum-conjugated bilirubin and total bilirubin levels (time points: the first day of birth, 1 week after birth, 2 weeks after birth).

**Results:**

(1) The changing trend of conjugated bilirubin was different between the two groups(*F =* 4.085, *P =* 0.019). The NEC group’s serum-conjugated bilirubin levels gradually increased ($\bar x$ ± *s*:12.64±2.68; 17.11±4.48; 19.25±11.63), while the non-NEC group did not show a continuous upward trend ($\bar x$ ± *s*:13.39±2.87; 15.63±3.75; 15.47±4.12). (2) Multiple analyses showed that patent ductus arteriosus(PDA) (*odds ratio*[*OR*] = 5.958, *95%confidence interval*[*CI*] = 2.102 ~ 16.882) and increased conjugated bilirubin in the 2nd week (*OR* = 1.105, *95%CI* = 1.013 ~ 1.206) after birth were independent risk factors for NEC.

**Conclusions:**

The body had already experienced an elevation of conjugated bilirubin before the occurrence of NEC. The change of early conjugated bilirubin may be an important factor in the occurrence of NEC.

## Background

Neonatal necrotizing enterocolitis (NEC) is a common critical illness of the gastrointestinal system in neonatal intensive care units, which manifests as ulceration, ischemia, and even necrosis of the intestinal wall tissue. Among neonates younger than 34 weeks in neonatal intensive care units in China, the incidence of NEC is about 3% [1]. NEC is prone to serious complications such as intestinal stricture, short bowel syndrome, and delayed growth and development.

In 1998, Mashall proposed the concept of the “gut-liver axis” [2]. The liver and gallbladder are connected with the intestine through the portal system, and intestinal microecology can affect the immune function of the liver [3]. On the one hand, when the intestine is damaged, the intestinal flora can transfer and enter the portal system; on the other hand, the flora and endotoxin entering the portal system will stimulate the liver macrophage system to release a series of inflammatory factors. This process aggravates the flora disturbance and causes intestinal mucosal damage. The intestinal and hepatobiliary systems form a complex network of interactions [4]. In neonatal bilirubin metabolism, there is special enterohepatic circulation. In this study, the clinical data of preterm infants were collected, and the differences in early serum- conjugated bilirubin between NEC preterm infants and non-NEC preterm infants were compared to understand the effect of serum-conjugated bilirubin on NEC.

## Methods

### Subjects and groups

Clinical data of premature infants were collected and retrospectively analyzed in the neonatal intensive care unit of the First Affiliated Hospital of Nanjing Medical University from 2017 to 2020. Inclusion criteria: (1) gestational age < 37 weeks; (2) hospitalized in neonatal department. Exclusion criteria: (1) Congenital intestinal malformations: such as Hirschsprung’s disease, intestinal atresia and chromosomal diseases, etc. (2) Congenital biliary malformations: absence of biliary tract, biliary atresia and genetic metabolic diseases; Renal disease and hepatitis cholestasis caused by bacterial and viral infections, etc. (3) Preterm infants with NEC occurring within 2 weeks of age (We want to explore the changing trend of conjugated bilirubin before the occurrence of NEC. Our department usually detects biochemical indicators once a week. For premature infants with NEC within 2 weeks, we cannot collect too many conjugated bilirubin indicators before the onset of the disease). (4) Early death, automatic discharge, and incomplete clinical data. Diagnostic criteria for NEC cases: according to the revised Bell staging [5]. There were 41 cases of NEC (stage IIA and above) diagnosed after 2 weeks of age, of which 2 cases were excluded due to incomplete data. Propensity-matching score (PSM) was used to adjust the gestational age, and birth weight between the groups (two of the children in the NEC group were not matched to the control group). Finally, 37 cases in the NEC group were included in the analysis (the average time to diagnosis was 18.9 days), and 74 cases were in the non-NEC group. The amount of feeding achieved at the time of diagnosis of NEC was low (about 20-30ml/kg.d). This study was approved by the Ethics Committee of the First Affiliated Hospital of Nanjing Medical University (2021SR439).

### Clinical characteristics

Gestational age; birth weight; small for gestational age: neonates with birth weight below the 10th percentile of birth weight for the same gestational age; gender; cesarean section(CS); patent ductus arteriosus(PDA): diagnosed by two-dimensional color Doppler examination in the first week of life and echocardiography indicating the presence of left-to-right shunts with a ductus arteriosus diameter > 1.5 mm and treated with ibuprofen; early anemia: defined as Hct < 39% within 1 week of life [6]; early infection: infection within 7 days after birth, including newborn sepsis, infectious pneumonia, etc.; neonatal respiratory distress syndrome(RDS); 1 and 5 min APGAR scores; mechanical ventilation and non-invasive ventilation; the first feeding time; feeding time of reaching half- milk; feeding time of reaching full- milk; type of milk; the serum-conjugated bilirubin (CB) and total bilirubin (TB): the first day of birth, 1 week after birth, and 2 weeks after birth; use of probiotics; feeding tolerance; Premature rupture of membranes; gestational diabetes; hypertension during pregnancy; chronic hypertension with pregnancy; liver damage or cholestasis during pregnancy and amniotic fluid contamination.

### Statistical analysis

We used SPSS software Version 26.0 for data analysis. Normally and approximately normally distributed data were expressed by $\bar x$ ± *s*, and the average number of two groups was compared with T-test. Data that did not conform to the normal distribution were represented by median (quarterfer distance), compared with U-test. Repetitive measurement changes were compared with repeated measurement variance analysis. The comparison of counting data adopted *χ2* test or Fisher accurate probability method. Multivariate regression analysis was used to analyze independent risk factors. The test criteria was *P <* 0.05. We used Graphpad Prism Version8.0 for the diagram.

## Results

### Relationship between early serum-conjugated bilirubin and NEC

#### Analysis of clinical data of the NEC group and the non-NEC group

We compared the clinical data of the two groups. The incidence of PDA was higher, and the time to start feeding and reaching half-feeding was longer in the NEC group. The difference was statistically significant. (Table 1)

**Table 1:** Comparison of clinical data between the two groups[*n(%);*$\bar x$* ± s; median (quarterfer distance)*]

	the NEC group *n* = 37	the non-NEC group *n* = 74	*z/t/x* ^*2*^	*P*	
**Infants**					
gestational age/w	29.15 ± 2.21	29.43 ± 1.98	-0.653	0.516	
birth weight/g	1267.84 ± 415.5	1318.11 ± 355.7	-0.630	0.531	
small for gestational age	8 (21.6)	14 (18.9)	0.113	0.736	
male	18 (48.6)	42 (56.8)	0.653	0.419	
APGAR(1 min)	5.84 ± 2.20	6.57 ± 2.36	-1.581	0.117	
APGAR(5 min)	7.24 ± 1.80	7.73 ± 1.65	-1.405	0.163	
mechanical ventilation (≥ 24 h)	20 (54.1)	32 (43.2)	1.158	0.282	
non-invasive ventilation	36 (97.3)	65 (87.8)	1.662	0.197	
probiotics	16 (43.2)	27 (36.5)	0.475	0.491	
first feeding time/d	7 (10)	4 (5)	2.294	0.022	
half feeding time/d	39.5 (22.5)	32(23)	2.925	0.003	
full feeding time/d	48 (24)	38 (27)	2.694	0.007	
type of milk(human milk)	25(67.6)	46(62.2)	0.313	0.576	
**complications**					
PDA	15 (40.5)	16 (21.6)	4.386	0.036	
anemia within 1 week	27 (73.0)	44 (59.5)	1.954	0.162	
infection within 1 week	29 (78.4)	46 (62.2)	2.960	0.085	
RDS	34 (91.9)	63 (88.7)	0.501	0.479	
feeding intolerance	14 (37.8)	20 (27.0)	1.357	0.244	
**maternal factors**					
gestational hypertension	7 (18.9)	12 (16.2)	0.127	0.722	
gestational diabetes	10 (27.0)	17 (23.0)	0.220	0.639	
liver damage or cholestasis during pregnancy	1 (2.7)	5 (6.8)	0.198	0.656	
premature rupture of membranes	10 (27.0)	30 (43.5)	1.954	0.162	
Amniotic fluid contamination	4 (10.8)	10 (13.5)	0.010	0.919	
cesarean section	18 (48.6)	33 (44.6)	0.163	0.686	

PDA: patent ductus arteriosus; RDS: respiratory distress syndrome

#### Comparison of early serum-conjugated bilirubin and total bilirubin between two groups

**Fig. 1:**
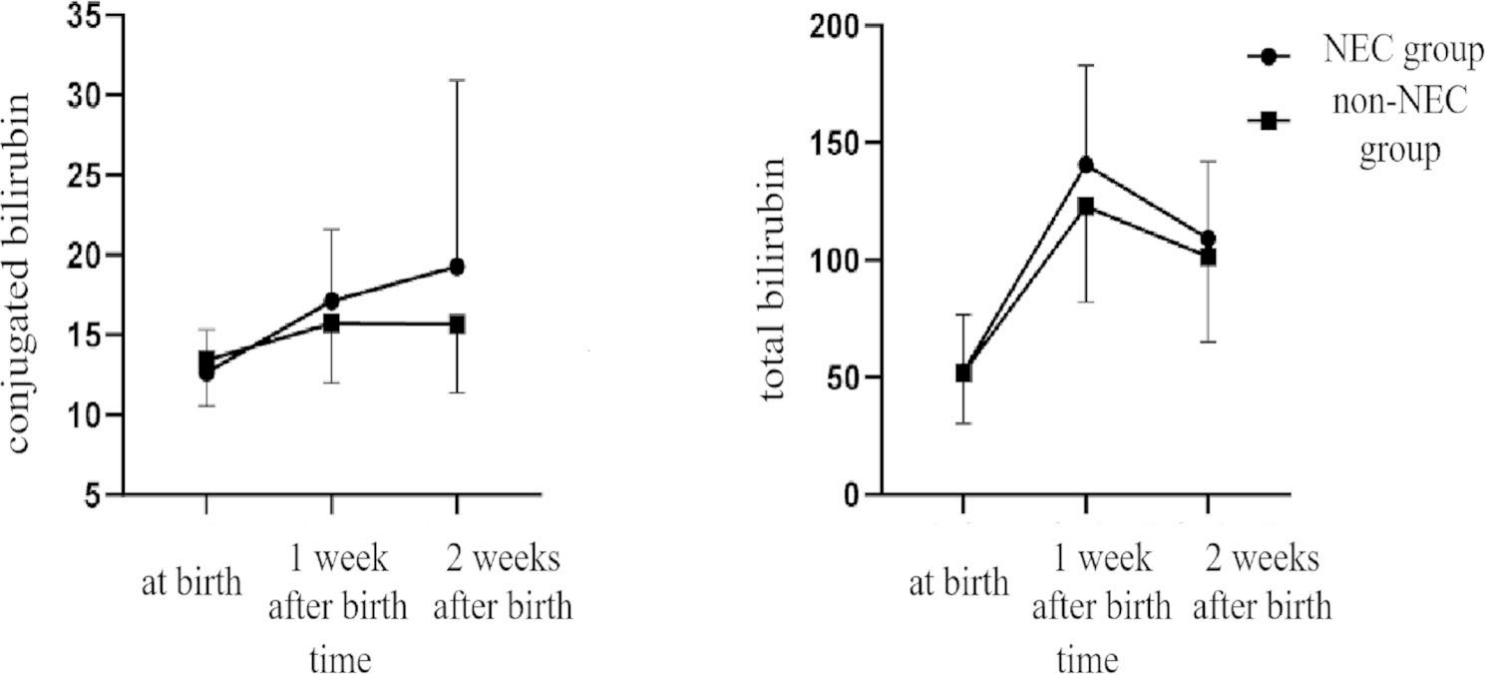
Changes of serum-conjugated bilirubin and serum-total bilirubin between two groups on the first day of birth, 1 week after birth, and 2 weeks after birth (umol/L)

Since the average diagnosis time of NEC in this study was 18.9 days, the serum conjugated bilirubin and total bilirubin values on the first day of life, 1 week after birth, and 2 weeks after birth were collected for analysis and comparison. There was an interaction between the serum-conjugated bilirubin value at different time points and the occurrence of NEC(P = 0.019). The changing trend of serum-conjugated bilirubin was different between the two groups (see Table 2; Fig. 1). The conjugated bilirubin showed a continuous upward trend in the NEC group and did not show the same trend in the non-NEC group. The changing trend of total bilirubin was also different between the two groups (P = 0.039) (see Table 2; Fig. 1). The total bilirubin at 1 week after birth and conjugated bilirubin 2 weeks after birth in the NEC group were higher than those in the non-NEC group. The difference was statistically significant.

**Table 2:** Comparison of serum bilirubin levels between two groups at different time points(umol/L)*(*$\bar x$* ± s)*

	the NEC group	the non-NEC group	t	p
CB at birth	12.64±2.68	13.39±2.87	-1.271	0.206
CB 1 week after birth	17.11±4.48	15.63±3.75	1.777	0.078
CB 2 weeks after birth	19.25±11.63	15.47±4.12	2.483	0.015
time*group	*F* = 4.085, *P* = 0.019			
TB at birth	52.27 ± 24.23	52.05 ± 21.63	0.046	0.963
TB 1 week after birth	140.56 ± 42.44	121.67 ± 39.52	2.269	0.025
TB 2 weeks after birth	109.15 ± 32.94	100.83 ± 35.71	1.173	0.243
time*group	*F* = 3.306 *P* = 0.039			

CB: conjugated bilirubin; TB: total bilirubin

#### Multivariate regression analysis

The influence factors of *P* < 0.1(first feeding time, half feeding time, full feeding time, PDA, infection within 1 week, CB 1 week after birth, CB 2 weeks after birth, TB 1 week after birth) in univariate analysis were included in multivariate regression. The results showed that PDA and increased serum-conjugated bilirubin at 2 weeks after birth were independent risk factors for the occurrence of NEC in preterm infants. The risk of NEC in children with PDA treated with ibuprofen was 5.958 times higher than that in children without PDA, and each unit of conjugated bilirubin increased the risk of NEC by 1.105 times. (Table 3)

**Table 3:** Multivariate logistic regression analysis

	*B*	*P*	*OR*	*95%CI*
PDA	1.785	0.001	5.958	2.102 ~ 16.882
CB (2 weeks after birth)	0.100	0.024	1.105	1.013 ~ 1.206
first feeding time	0.084	0.187	1.088	0.960 ~ 1.233
half feeding time	0.014	0.275	1.015	0.989 ~ 1.041
full feeding time	-0.026	0.353	0.974	0.922 ~ 1.029
infection within 1 week	0.150	0.796	1.162	0.372 ~ 3.629
CB (1 week after birth)	0.053	0.439	1.055	0.922 ~ 1.207
TB (1 week after birth)	0.007	0.265	1.007	0.995 ~ 1.020

PDA: patent ductus arteriosus; CB: conjugated bilirubin; TB: total bilirubin

## Discussions

NEC is more common in preterm and low birth weight infants than full-term babies [7], in addition, preterm infants usually develop this disease at 2–4 weeks after birth. Symptoms of NEC are often atypical, which makes the early diagnosis of NEC difficult. Therefore clinicians may miss the opportunity for diagnosis and treatment, which leads to an increase in surgical rates. In this study, we included a variety of prenatal and postnatal factors and found that feeding time, PDA, and bilirubin were associated with the occurrence of NEC. Through multivariate analysis, we found that PDA and increased conjugated bilirubin in the 2nd week after birth were associated with NEC. Conjugated bilirubin showed a continuous upward trend in the NEC group before the occurrence of NEC, while the non-NEC group did not show this trend, which is a new finding compared to previous studies. We think that the increased serum-conjugated bilirubin before the occurrence of NEC may have an impact on the occurrence of NEC. The following points are possible mechanisms. (1) Impaired gut physical barrier: Sustained elevation of conjugated bilirubin may indicate the occurrence of cholestasis, where bile acid secretion may also be impaired. A study has shown that normal physiological doses of bile salts excreting into the intestine can promote the proliferation of intestinal mucosal epithelial cells [8]. An animal study [9]showed that in a murine model of cholestasis, the small intestinal mucosa was edematous, the distribution of epithelial tight junction proteins was abnormal, and the permeability of intestinal mucosa was increased. (2) Transfer of intestinal flora: Conjugated bilirubin secreted from the liver into the bile flows to the intestine for excretion. It undergoes microbial catabolism in the intestine and converts to urobilinogen, then forms stercobilinogen for excretion. Elevated serum-conjugated bilirubin may indicate changes in the gut microbiome. Animal studies have shown that in mice with cholestasis, coupled with elevated bilirubin, the diversity of intestinal flora is lower than that in normal mice [10]. (3) Hormonal changes: Decreased bile secretion affects the normal secretion of gastrointestinal hormones [11], resulting in digestive tract dysfunction. These factors accelerate intestinal damage, which may lead to neonatal necrotizing enterocolitis. To sum up, the increase of conjugated bilirubin may cause certain intestinal damage.

In addition, before the diagnosis of NEC, some pathological damage may have occurred in the intestine. The intestinal mucosa is damaged, and gastrointestinal hormones such as cholecystokinin are reduced. This process may affect the normal excretion of bile, resulting in an increase of conjugated bilirubin. The diversity of gut microbiota decreased [12]. When the intestinal flora decreases, the intestinal environment can be alkaline, and the activity of β-glucuronidase increases in this environment [13]. The enterohepatic circulation of bilirubin is active, and more bilirubin is returned to the blood through the portal vein. As a result, serum bilirubin rises before diagnosis of NEC.

We also found that a delay in the time of first milk initiation may affect the occurrence of NEC, but it was not an independent risk factor for NEC. This is similar to the study by Kimak, who found that preterm infants who started breastfeeding for more than 5 days had an increased incidence of NEC compared with preterm infants who started breastfeeding 1 day after birth [14]. Previous studies have shown that delayed feeding was not associated with an increased risk of NEC [15, 16]. A recent Cochrane meta-analysis found that in very preterm and low-birth-weight infants, delayed feeding may slightly reduce feeding intolerance, but at the same time may increase the risk of invasive infection [17]. For premature and low-birth-weight infants, whether enteral feeding should be started as early as possible remains to be discussed.

At the same time, this study showed that PDA was an independent risk factor for NEC. Ndour et al. thought those preterm infants with hemodynamically significant patent ductus arteriosus(hsPDA) and treated with oral ibuprofen within 1 week were prone to gastrointestinal complications (NEC and intestinal perforation) [18]. This is consistent with our conclusion. A meta-analysis found that hsPDA was an independent risk factor for NEC with or without medication [19]. Salvatori et al. found that hypoperfusion due to PDA can influence nutrition because of malabsorption, feeding intolerance or intestinal ischemia [20]. Intestinal microcirculation disorders are one of the factors that cause intestinal necrosis in NEC. PDA affects blood flow, so hsPDA may cause NEC [21].

Since this study is a retrospective study, there are many clinical confounding factors, and the results are limited. In the future, it is necessary to expand the sample size and carry out mechanism research to predict the occurrence and development of the disease.

## Conclusions

To sum up, before the diagnosis of NEC, the body may have experienced changes in serum-conjugated bilirubin, showing a continuously increasing trend. The changes in early conjugated bilirubin may be an important indicator for the occurrence of NEC in premature infants. It may provide new ideas for the pathogenesis of NEC.

## Data Availability

The datasets used and/or analysed during the current study are available from the corresponding author on reasonable request.

## List of abbreviations

NEC: necrotizing enterocolitis
PSM: propensity-matching score
PDA: patent ductus arteriosus
CS: cesarean section
RDS: respiratory distress syndrome
CB: conjugated bilirubin
TB: total bilirubin
hsPDA: hemodynamically significant patent ductus arteriosus
